# Fast estimation of adult cerebral blood content and oxygenation with hyperspectral time-resolved near-infrared spectroscopy

**DOI:** 10.3389/fnins.2023.1020151

**Published:** 2023-02-16

**Authors:** David Jonathan Fulop Cohen, Natalie C. Li, Seva Ioussoufovitch, Mamadou Diop

**Affiliations:** ^1^Department of Medical Biophysics, Western University, London, ON, Canada; ^2^School of Biomedical Engineering, Western University, London, ON, Canada; ^3^Imaging Program, Lawson Health Research Institute, London, ON, Canada

**Keywords:** near-infrared spectroscopy, hyperspectral time-resolved, neuromonitoring, oxygen saturation, adult, NIRS, real-time, two-layer model of the adult head

## Abstract

Near-infrared spectroscopy (NIRS) can measure tissue blood content and oxygenation; however, its use for adult neuromonitoring is challenging due to significant contamination from their thick extracerebral layers (ECL; primarily scalp and skull). This report presents a fast method for accurate estimation of adult cerebral blood content and oxygenation from hyperspectral time resolved NIRS (trNIRS) data. A two-phase fitting method, based on a two-layer head model (ECL and brain), was developed. Phase 1 uses spectral constraints to accurately estimate the baseline blood content and oxygenation in both layers, which are then used by Phase 2 to correct for the ECL contamination of the late-arriving photons. The method was validated with *in silico* data from Monte-Carlo simulations of hyperspectral trNIRS in a realistic model of the adult head obtained from a high-resolution MRI. Phase 1 recovered cerebral blood oxygenation and total hemoglobin with an accuracy of 2.7 ± 2.5 and 2.8 ± 1.8%, respectively, with unknown ECL thickness, and 1.5 ± 1.4 and 1.7 ± 1.1% when the ECL thickness was known. Phase 2 recovered these parameters with an accuracy of 1.5 ± 1.5 and 3.1 ± 0.9%, respectively. Future work will include further validation in tissue-mimicking phantoms with various top layer thicknesses and in a pig model of the adult head before human applications.

## 1. Introduction

Near-infrared spectroscopy (NIRS) is a portable technology that uses safe (i.e., non-ionizing) near-infrared light to noninvasively probe living tissue (Denault et al., 2018). NIRS has high sensitivity to key biomarkers of brain health such as cerebral blood content and oxygenation (Durduran et al., 2010), and is now widely used for neuromonitoring in both pre-clinical and clinical settings (Brown et al., 2002; Goldman et al., 2004; Lewis et al., 2018; Abdalmalak et al., 2020a,b; Milej et al., 2020b). Several NIRS methods have been developed over the years, but the most popular are based on continuous-wave NIRS (cwNIRS), which is the simplest NIRS technology and is based on sending a light beam of constant intensity into the tissue and monitoring for changes in light attenuation (Scholkmann et al., 2014; Wojtkiewicz et al., 2014). Importantly, by using a cwNIRS technique that can measure light attenuation at dozens of wavelengths, rather than just a few, the spectral features of the main light absorbers in tissue (e.g., water, oxyhemoglobin and deoxyhemoglobin) can be leveraged to improve the accuracy of cwNIRS (Diop et al., 2014). This approach is often called *hyperspectral* cwNIRS and has been shown to be reliable for neonatal neuromonitoring; (Diop et al., 2014; Rajaram et al., 2018) however, hyperspectral cwNIRS neuromonitoring in adults remains a challenge due to significant contamination from their thicker extracerebral layers (ECL; scalp, skull, and cerebrospinal fluid) (Elliott et al., 2010; Milej et al., 2020a; Li and Diop, 2022).

To mitigate this challenge, alternative NIRS methods that are more sensitive to deep-lying tissue have been developed, and the most advanced are based on time-resolved NIRS (trNIRS) (Gagnon et al., 2008; Diop et al., 2010). In trNIRS neuromonitoring, short pulses of light are released into the head, and the arrival time of each photon at the detector is precisely measured to generate a distribution of time-of-flight (DTOF). This allows for the differentiation of early arriving photons, which have only passed through the ECL, from late-arriving photons which are more likely to probe the brain. Several studies have demonstrated the superior brain sensitivity of trNIRS; (Chance et al., 1988; Liebert et al., 2003; Selb et al., 2005; Steinbrink et al., 2006) however, analyzing trNIRS brain measurements is challenging. Consequently, measurements are often interpreted by assuming that the brain is a homogeneous medium (Patterson et al., 1989; Gerega et al., 2018; Baker et al., 2019). While such an approach may work in neonates, given their thin (3–5 mm) ECL (Li et al., 2015; Sharma et al., 2020), modeling the adult head as a homogeneous medium is an oversimplification since NIRS probes are typically positioned on the scalp for non-invasive measurements. As such, adult measurements contain significant contribution from the ECL since light must travel through skin (∼1.2–1.5 mm) and thick skull (∼10 mm) before reaching the brain (Selb et al., 2014b).

A simple, yet more accurate, approach to account for the ECL contribution is to divide the head into two compartments: brain and ECL (Gagnon et al., 2008). This permits the use of analytical solutions of light propagation in two-layer turbid media to analyze adult trNIRS brain measurements (Kienle et al., 1998). Nevertheless, analyzing trNIRS data with a two-layer analytical model is prone to crosstalk because of the increased number of fitting parameters (from 3 for a homogeneous medium, to 6): 4 parameters for the absorption and scattering coefficients of both layers, the thickness of the top layer, and an amplitude term that accounts for the unknown gain of the trNIRS system. We hypothesize that trNIRS data at dozens of wavelengths, similar to hyperspectral cwNIRS, will allow for the use of the spectral features of tissue chromophores to better constrain the fitting and reduce crosstalk.

To test this hypothesis, we developed a two-phase fitting algorithm−based on a two-layer analytical model of light propagation in diffuse media (Kienle et al., 1998)−that leverages the spectral features of oxyhemoglobin and deoxyhemoglobin to estimate their concentration in the adult brain from hyperspectral trNIRS data acquired with probes positioned on the scalp. Phase 1 of the algorithm uses the two-layer analytical model to accurately estimate initial (i.e., baseline) chromophore concentrations, and Phase 2 uses these initial values and late-photon analysis (i.e., the tail of the DTOFs) to rapidly estimate subsequent concentrations. This approach significantly reduces computation time while accounting for the ECL contribution to the optical signal. The accuracy of the method was validated with *in silico* data from Monte-Carlo simulations of hyperspectral trNIRS in a realistic model of the adult head obtained from a high-resolution MRI. The estimated concentrations were compared with the known inputted values using Pearson’s correlation and Bland-Altman plots.

## 2. Materials and methods

**FIGURE 1:**
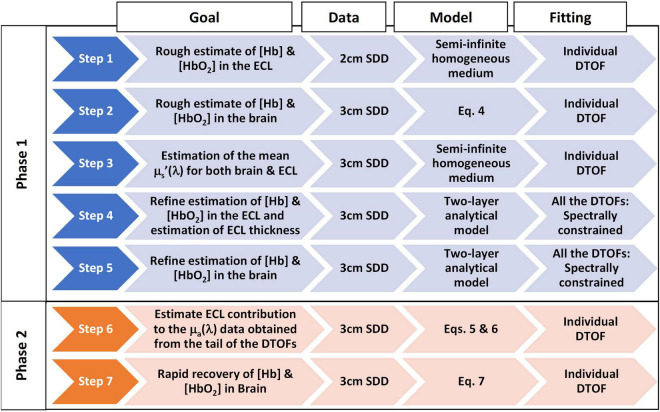
Flowchart of the two-phase hyperspectral trNIRS data analysis algorithm. The following provides additional information on each step of the algorithm. Step 1: 1st fitting: Fitted for Amplitude, μ_a_(λ), and μ_s_’(λ); 2nd fitting: Amplitude was fixed to the mean value obtained in the 1st fitting and fitted for μ_a_(λ) and μ_s_’(λ); 3rd fitting: Amplitude and μ_s_’(λ) were fixed to the values obtained in the 1st and 2nd fitting, and fitted for μ_a_(λ) only; Computed [Hb] and [HbO_2_] from μ_a_(λ). Step 2: The tail of the logarithm of the DTOFs was fit to obtain μ_a_(λ); Computed [Hb] and [HbO_2_] from μ_a_(λ). Step 3: 1st fitting: Fitted for Amplitude, μ_a_(λ), and μ_s_’(λ); 2nd fitting: Amplitude was fixed and fitted for μ_a_(λ), and μ_s_’(λ); the latter were used as the mean reduced scattering coefficient for both brain and ECL. Step 4: [Hb] and [HbO_2_] in the brain were fixed to the values obtained in Step 2; μ_s_’(λ) fixed to values obtained in Step 3; Fitting parameters: ECL thickness, and [Hb] and [HbO_2_] in the ECL; The initial guess of [Hb] and [HbO_2_] were the values obtained in Step 1 and were allowed to vary ± 50%; ECL thickness was set to the average value obtained from the segmented MRI and allowed to vary ± 10%. Step 5: [Hb] and [HbO_2_] in the ECL were fixed to the values obtained in Step 4; μ_s_’(λ) fixed to values obtained in Step 3; Fitting parameters: [Hb] and [HbO_2_] in the brain; The initial guess of [Hb] and [HbO_2_] in the brain was 30 μM and allowed to vary between 0 and 80 μM. Step 6: The absorption coefficients in the ECL, *μ_a_ECL__* (λ), and the brain, *μ_a_Brain__* (λ), obtained from Steps 4 and 5 were used to estimate the ECL contribution (f_ECL_) to the absorption coefficients from the late-photon analysis, *μ_a_Lp__* (λ). Step 7: For any subsequent trNIRS spectrum (i.e., DTOFs dataset), the absorption coefficient in the brain, *μ_a_Brain__* (T, λ), can be rapidly estimated from the baseline absorption coefficients in the brain and ECL, and the ECL contribution to the absorption coefficients obtained from late photon analysis of the DTOFs (f_ECL_).

The different steps of the two-phase fitting algorithm are illustrated in Figure 1. In Phase 1, data from two source-detector distances (2 and 3 cm) are fit separately with a solution to the diffusion approximation (DA) for a semi-infinite two-layer medium to recover the *absolute* concentrations of oxyhemoglobin and deoxyhemoglobin in the brain (i.e., baseline concentrations) (Kienle et al., 1998). Phase 2 focuses on rapid estimation of the brain chromophore concentrations once their baseline values are known. It is noteworthy that Phase 2 uses data from both source-detector distances to account for the ECL contribution to the signal.

### 2.1. Phase 1: Estimation of baseline chromophore concentrations

There are typically six fitting parameters when a trNIRS curve (i.e., DTOF) is analyzed with an analytical model of light transport in a two-layer diffuse medium: the scattering and absorption coefficients of each layer, the top layer thickness, and an amplitude term. Because of the large number of fitting parameters, such a procedure is prone to crosstalk between the parameters (Gagnon et al., 2008). The current algorithm reduces crosstalk by dividing the fitting procedure into five steps: (i) roughly estimate the ECL chromophore concentrations, (ii) roughly estimate the brain chromophore concentrations, (iii) estimate the scattering coefficients, (iv) refine the ECL concentrations, and (v) refine the brain concentrations. Further, the algorithm leverages the spectral content of the data to better constrain the fitting, thereby further reducing crosstalk. The details of each step are provided in the following sections.

#### 2.1.1. Step 1: Rough estimation of the concentrations of oxyhemoglobin and deoxyhemoglobin in the ECL

The goal of this step is to use the shorter (2 cm) source-detector distance data to obtain rough estimates of the concentrations of oxyhemoglobin and deoxyhemoglobin in the ECL, which will be further refined in Step 4. Since more than 80% of the signal obtained at the 2 cm source-detector distance comes from the ECL (Milej et al., 2020a), analyzing this data with a solution to the DA for a semi-infinite *homogeneous* medium will yield results that are heavily weighted toward the ECL parameters. The homogeneous medium fitting was implemented in MATLAB 2020B using a bounded least-squares regression method [*fminsearchbnd* (D’Errico, 2021)] to minimize the difference between the analytical model of light propagation in a semi-infinite homogeneous medium and the DTOF data at each wavelength. The DTOFs were fit from 50% of the max on the leading edge to 5% of the max after the peak. The three fitting parameters in the homogeneous fitting were the absorption coefficient, reduced scattering coefficient, and amplitude. The 2 cm data were analyzed through three rounds of fitting, with all three parameters allowed to vary freely in the first round. Thereafter, the mean amplitude for all wavelengths was computed, and the DTOFs were fit a second time with the amplitude fixed to this mean value, to obtain estimates of the wavelength-dependent absorption and reduced scattering coefficients. The wavelength-dependent scattering coefficients recovered from this round were then fit to a model of Mie Scattering (Eq. 1) to further reduce the noise in the estimated scattering coefficients. Note that in Eq. 1, *a* and *b* are the scattering amplitude and power, respectively, λ is the wavelength in nm, and μ′s⁢(λ) is the wavelength-dependent reduced scattering coefficient (*mm*^−1^).


(1) μ′s⁢(λ)=a⁢(λ800)-b

**FIGURE 2:**
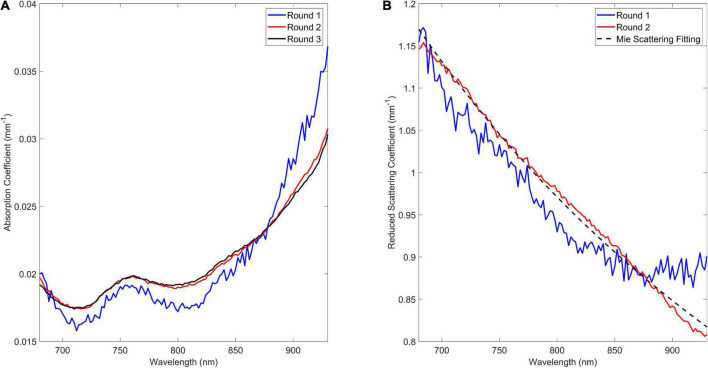
Wavelength-dependent absorption **(A)** and reduced scattering **(B)** coefficient obtained following each round of fitting the 2 cm distance data assuming a homogenous medium.

Thereafter, the DTOFs are fit a third time–with both the mean amplitude and reduced scattering values fixed to their values obtained from the previous round of fitting–to estimate the wavelength-dependent absorption coefficient (Figure 2).

The absorption coefficient depends on the tissue chromophores’ concentrations (*C_i_*) and their wavelength-dependent extinction coefficients (ε_*i*_):


(2) $$\mu_{a}\,\left(\lambda \right)=\sum_{i}C_{i}\,\varepsilon_{i}\,\left(\lambda \right)$$

This relationship can be used to recover the concentrations of oxyhemoglobin (HbO_2_) and deoxyhemoglobin (Hb) from the wavelength-dependent absorption coefficients by solving the following system of linear equations (Eq. 3):


(3) $$\begin{array}{c} \mu_{a}\,\left(\lambda_{1}\right)\\ \begin{array}{c} \mu_{a}\,\left(\lambda_{2}\right)\\ \vdots \end{array}\\ \mu_{a}\,\left(\lambda_{n}\right) \end{array}=\begin{array}{c} W\,F\,\varepsilon_{W\,a\,t\,e\,r}\,\left(\lambda_{1}\right)+C_{H\,b}\,\varepsilon_{H\,b}\,\left(\lambda_{1}\right)+C_{H\,b\,O_{2}}\,\varepsilon_{H\,b\,O_{2}}\,\left(\lambda_{1}\right)\\ \begin{array}{c} W\,F\,\varepsilon_{W\,a\,t\,e\,r}\,\left(\lambda_{2}\right)+C_{H\,b}\,\varepsilon_{H\,b}\,\left(\lambda_{2}\right)+C_{H\,b\,O_{2}}\,\varepsilon_{H\,b\,O_{2}}\,\left(\lambda_{2}\right)\\ \vdots \end{array}\\ W\,F\,\varepsilon_{W\,a\,t\,e\,r}\,\left(\lambda_{n}\right)+C_{H\,b}\,\varepsilon_{H\,b}\,\left(\lambda_{n}\right)+C_{H\,b\,O_{2}}\,\varepsilon_{H\,b\,O_{2}}\,\left(\lambda_{n}\right) \end{array}$$

where WF is the water fraction and is assumed to be 80% (Oros-Peusquens et al., 2019).

#### 2.1.2. Step 2: Rough estimation of the concentration of oxyhemoglobin and deoxyhemoglobin in the brain

Step 2 establishes preliminary rough estimates of the concentrations of oxyhemoglobin and deoxyhemoglobin in the brain. One of the benefits of using trNIRS is that the tail of the DTOF is highly sensitive to deep tissue absorption (Diop and Lawrence, 2013). As such, the tail of the DTOFs were used to obtain a rough estimation of the brain absorption coefficient:


(4) $$\mu_{a}\,\left(\lambda \right)≅-\frac{\frac{\partial \text{ln}\,\left(D\,T\,O\,F\,\left(\lambda ,t\right)\right)}{\partial t}}{\frac{c}{n}}$$

where *c* is the speed of light in a vacuum, and *n* is the refractive index of the tissue (assumed to be equal to 1.4). The tail of the DTOFs were fit within the region between 5 and 1% of the max after the peak. All wavelengths from the long source-detector distance (3 cm) were individually analyzed using Eq. 4. The absorption coefficients were used to compute the rough initial estimates of oxyhemoglobin and deoxyhemoglobin concentrations in the brain, similar to Eq. 3. These concentrations will be later used to constrain the fitting of the 3 cm data with the two-layer analytical model (Step 4).

#### 2.1.3. Step 3: Estimation of the reduced scattering coefficient

Step 3 analyzes the DTOFs from the 3 cm source-detector distance, using the process outlined in Step 1, to estimate the mean reduced scattering coefficient of the combined two-layer (i.e., both the ECL and the brain). Note that we are assuming homogenous tissue scattering, instead of layer-specific scattering coefficients, as this reduces both the duration and complexity of the fitting.

#### 2.1.4. Step 4: Refining the extracerebral concentrations

In Step 4, the estimates of oxyhemoglobin and deoxyhemoglobin concentrations in the ECL are refined by *simultaneously* fitting the DTOFs of all the wavelengths with an analytical model of light transport in a two-layer diffusive medium (Kienle et al., 1998). The fitting parameters for this step are the concentrations of oxyhemoglobin and deoxyhemoglobin in both layers and the ECL thickness. The fitting algorithm was implemented in MATLAB, using the function *fminsearchbnd* to minimize the difference between the analytical model and the DTOFs of the full spectrum.

In this step, the concentrations of oxyhemoglobin and deoxyhemoglobin in the brain are fixed to the values obtained in Step 2, ECL concentrations estimated in Step 1 are used as the initial guess and allowed to vary ± 50%, and the ECL thickness is assigned an initial value equal to the average from the segmented MRI and allowed to vary ± 10%. The reduced scattering coefficients were fixed to the values obtained in Step 3. Finally, the amplitude is assigned by multiplying the one-layer amplitude from the homogenous fitting in Step 3 with the quotient of the one-layer solution divided by the two-layer solution, using the homogenous optical properties from Step 3. This corrects for any discrepancy in the amplitude of the two solutions. The fitting was conducted on the 3 cm source-detector distance DTOFs from 50% of the maximum on the leading edge to 5% after the peak. The refined ECL concentrations and thickness are then used in Step 5 to refine the brain concentrations.

#### 2.1.5. Step 5: Refining the cerebral concentrations

In the final step of Phase 1, the ECL parameters recovered from Step 4 are fixed, and the concentrations of oxyhemoglobin and deoxyhemoglobin in the brain “layer” are allowed to vary between 0 and 80 *μ*M, with an initial value of 30 *μ*M. Note that the rough estimates of brain chromophores’ concentrations obtained in Step 2 could be used as initial guess and allowed to vary ± 50%. However, because this step is very stable once the scattering coefficient, the ECL thickness, and the concentrations of the chromophores in the ECL are determined (Step 3 and 4), we relaxed the constraints for the concentrations of oxyhemoglobin and deoxyhemoglobin to illustrate the flexibility of the algorithm. The DTOFs obtained at the 3 cm source-detector distance for all wavelengths were simultaneously fit using the two-layer analytical model, from 5 to 1% of the maximum on the tail edge of the curves. This choice was guided by the fact late-arriving photons (i.e., the tail of the DTOF) are more sensitive to deep-lying tissue which, in this case, represents the brain layer. The estimated concentrations represent the baseline brain concentrations of oxyhemoglobin and deoxyhemoglobin, which are used in Phase 2 for rapid estimation of subsequent cerebral blood content and oxygenation.

### 2.2. Phase 2: Rapid estimation of cerebral blood content and oxygenation

Phase 2 uses the absorption coefficients estimated using the late-photon analysis (Eq. 4) on the 3 cm source-detector data to correct for the ECL contamination. This is accomplished by leveraging the absolute concentrations of the chromophores in both layers measured in Phase 1 (Eq. 5). Phase 2 has two steps: estimation of the ECL contribution to the absorption coefficient computed using late-photon analysis of the 3 cm SDD data (Step 6), and rapid estimation of chromophore concentrations in the brain (Step 7). Step 7 can be repeated for all subsequent datasets (i.e., spectrum of DTOFs), enabling rapid neuromonitoring (∼0.18 s per analysis). Note that while Phase 1 is compatible with a spectral resolution of 10 nm, Phase 2 requires at least a spectral resolution of 2 nm between 680 and 930 nm. The increased spectral resolution is needed for the late-photon analysis due to the reduced signal-to-noise ratio (SNR) of late arriving photons.

#### 2.2.1. Step 6: Estimation of the extracerebral contribution

Step 6 estimates the contribution of the ECL to the absorption coefficients recovered from the late-photon analysis of the DTOFs from the 3 cm SDD. This allows the fitting algorithm to account for the ECL contamination in subsequent hyperspectral trNIRS datasets, without the need to repeat the more computationally intensive two-layer analysis. More specifically, the absorption coefficients obtained from the late-photon analysis, *μ_a_LP__* (*t*, λ), can be expressed as a weighted average of the absorption coefficients of the two layers: (Jacques, 2013).


(5) $$\mu_{a_{L\,P}}\left(T=0,\lambda \right)=f_{E\,C\,L}\left(\lambda \right)\times \mu_{a_{E\,C\,L}}\left(T=0,\lambda \right)+$$

$$\;f_{B\,r\,a\,i\,n}\left(\lambda \right)\times \mu_{a_{B\,r\,a\,i\,n}}\left(T=0,\lambda \right)$$

where *μ_a_Brain__*(*T* = 0, λ) and *μ_a_ECL__*(*T* = 0, λ) are the baseline brain and ECL absorption coefficients computed by inputting the absolute concentrations estimated in Step 4 and 5 into Eq. 2, and assuming 80% water concentration. *μ_a_LP__*(*T* = 0, λ) are the absorption coefficients recovered by applying the late-photon analysis to the baseline DTOFs from the 3 cm SDD.

In a two-layer head model, the contribution of one layer can be expressed as a function of the other, such as *f*_*Brain*_ = 1 − *f*_*ECL*_, where *f*_*Brain*_ is the fraction of the absorption coefficient coming from the brain layer and *f*_*ECL*_ is the fraction from the ECL. Thus, Eq. 5 can be rearranged to isolate the ECL fraction:


(6) $$f_{E\,C\,L}\,\left(\lambda \right)=\frac{\mu_{a_{L\,P}}\left(T=0,\lambda \right)-\mu_{a_{B\,r\,a\,i\,n}}\left(T=0,\lambda \right)}{\mu_{a_{E\,C\,L}}\left(T=0,\lambda \right)-\mu_{a_{B\,r\,a\,i\,n}}\left(T=0,\lambda \right)}$$

#### 2.2.2. Step 7: Rapid estimation of deep tissue chromophore concentration

Step 7 allows rapid estimation of oxyhemoglobin and deoxyhemoglobin concentrations in the bottom layer (i.e., the brain). By combining Eqs. 5 and 6, the time-dependent absorption coefficient in the brain, *μ_a_Brain__* (*T*, λ), can be expressed as a function of the baseline absorption coefficients in each layer, *f*_*ECL*_, and the baseline late-photon absorption coefficient:


(7) $$\;\mu_{a_{B\,r\,a\,i\,n}}\,\left(T,\lambda \right)=$$

$$\mu_{a_{B\,r\,a\,i\,n}}\left(\text{T}=0,\lambda \right)*\left(1+\frac{\mu_{a_{L\,P}}\left(T,\lambda \right)-\mu_{a_{L\,P}}\left(T=0,\lambda \right)}{\mu_{a_{L\,P}}\left(T=0,\lambda \right)-f_{E\,C\,L}\left(\lambda \right)\mu_{a_{E\,C\,L}}\left(T0,\lambda \right)}\right)$$

Furthermore, using Eq. 2 and assuming a water concentration of 80%, the wavelength-dependent absorption coefficient can be used to compute the concentrations of oxyhemoglobin and deoxyhemoglobin in the brain. Cerebral oxygen saturation (*SO*_2_) and total cerebral hemoglobin (*HbT*) are then computed using Eqs. 8 and 9, respectively:


(8) $$S\,O_{2}=\frac{C_{H\,b\,O_{2}}}{C_{H\,b\,O_{2}}+C_{H\,b}}$$

(9) $$H\,b\,T=C_{H\,b\,O_{2}}+C_{H\,b}$$

### 2.3. Validation

**FIGURE 3:**
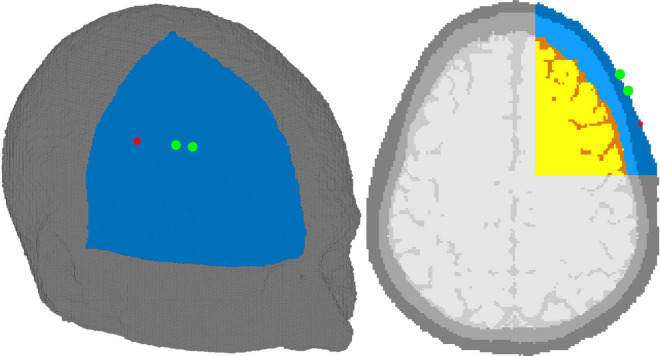
Adult head model with source (red dot) and detectors (green dot). The source-detector distances were 2 and 3 cm. The tissues shown are skin (dark blue), skull (light blue), cerebrospinal fluid (orange), and brain (yellow). Due to GPU limitations, only the right upper octant of the head was simulated and is shown above–gray tissues not included in the simulations.

Validation was conducted using *in silico* data generated with Monte-Carlo Extreme (MCX) in an adult head model at various skin and brain oxygen saturations (Fang and Boas, 2009; Yan and Fang, 2020). Using *in silico* data allows for direct comparison of the results of the algorithm with the “ground truth” inputted parameters while maintaining realistic geometry and optical properties. To better mimic experimental conditions, we segmented an MRI of an adult head into four tissue types: scalp, skull, cerebrospinal fluid (CSF), and brain using 3DSlicer (Kikinis et al., 2014). Brain and scalp oxygen saturations were varied independently from 40 to 80% and 50 to 70%, respectively, in 2% increments. The wide range of scalp oxygen saturations were simulated to investigate the confounding effects of changing scalp SO_2_ on the accuracy of estimating brain SO*_2_*. 126 simulations were conducted for each brain-skin pair, corresponding to the wavelength range of 680 to 930 nm, in 2 nm increments. In total, we completed 8,316 simulations for this validation. The source was positioned on the right side of the head with the detectors placed 2 and 3 cm toward the forehead, as shown in Figure 3. Each simulation had a total of 3 billion photons with random seeds, ensuring realistic photon statistics with high SNR at both detectors. The optical properties of bone and CSF, the scattering coefficient, anisotropy factor, and refractive indices of the skin and brain, were obtained from literature (Firbank et al., 1993; Jacques, 2013). Absorption coefficients of skin and brain were computed using Eq. 2 for each oxygen saturation in the aforementioned range. Total hemoglobin in the skin and brain were set to 12.4 and 55 *μ*Mol, respectively, for all the simulations (Luttkus et al., 1995; Kanti et al., 2014; Auger et al., 2016; Farzam et al., 2017).

## 3. Results

Estimation of the baseline chromophore concentrations from a full spectrum of DTOFs (i.e., Phase 1) required 315 s of computation (CPU: Intel Core i7-6800K @ 3.4 GHz, using parallel computing with 6 cores, GPU: EVGA NVIDIA GEFORCE GTX 1080 8 GB, RAM: 4 Kingston HyperX 32 GB, totaling 128 GB), while Phase 2 only took 0.18 s.

### 3.1. Phase 1: Baseline chromophore concentrations

**FIGURE 4:**
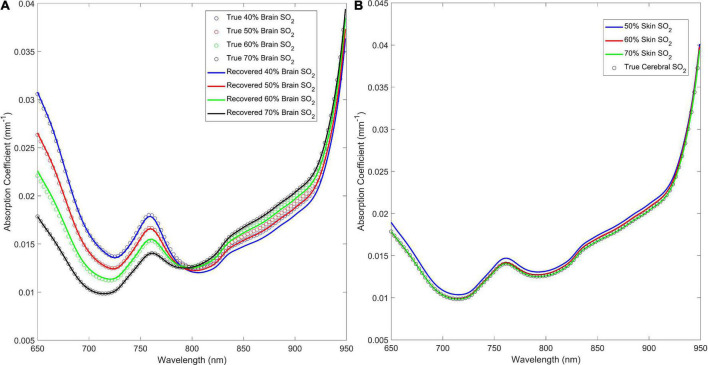
**(A)** Estimated absorption coefficient for the 40, 50, 60, and 70% brain oxygen saturations with skin oxygen saturation set to 70% versus the true (inputted) values. **(B)** Recovered versus true (inputted) absorption coefficient spectra for 50, 60, and 70% skin oxygen saturation at 70% brain oxygen saturation.

The estimated absorption spectra and the expected wavelength-dependent absorption for four brain and three skin oxygen saturations are shown in Figure 4 to illustrate the qualitative similarity between the recovered and “ground truth” absorption spectra. Figure 4 shows that when cerebral oxygen saturation decreases, the error in the recovered absorption spectrum increases slightly; this is particularly obvious above 800 nm and is a result of errors in the recovered oxyhemoglobin concentration (Figure 4A). Further, Figure 4B shows that the algorithm has excellent sensitivity to the brain as the ECL oxygen saturation has negligible impact on the accuracy of the estimated cerebral absorption coefficient.

**FIGURE 5:**
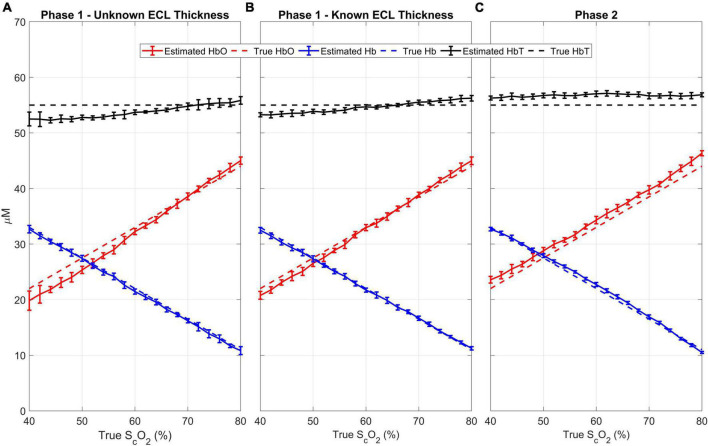
Results from Phase 1 with unknown **(A)** and known **(B)** ECL thickness and Phase 2 **(C)**: For every cerebral oxygen saturation, 11 scalp saturations (50 to 70 in 2% increments) were evaluated. The estimated oxyhemoglobin (red), deoxyhemoglobin (blue), and total hemoglobin (black) are plotted against the true cerebral SO2. The dotted lines are the “ground truth” while the solid lines are the estimated values. The error bars represent the standard deviation.

The results of the quantitative analysis are shown in Figure 5A. The mean (± standard deviation) difference between the recovered brain oxygen saturations and the true values is 2.7 ± 2.5%, and the correlation coefficient between the true and recovered values is 0.99 (*p* < 0.0001). For the total hemoglobin, the mean (± standard deviation) difference between the recovered concentration and the simulated concentration of 55 μM is 2.8 ± 1.8%. The mean ECL thickness was estimated to be 12.4 mm, representing a mean (± standard deviation) difference of 3.0 ± 2.2% from the “ground truth.”

For comparison, Phase 1 was repeated with the ECL thickness fixed to its known value of 12 mm, which led to more accurate estimates of all the hemodynamic parameters (Figure 5B). Notably, the mean (± standard deviation) difference between the recovered brain oxygen saturation was reduced to 1.5 ± 1.4%, with a Pearson’s correlation coefficient of 0.99 (*p* < 0.0001), and the total hemoglobin was recovered with a mean (± standard deviation) difference of 1.7 ± 1.1%.

**FIGURE 6:**
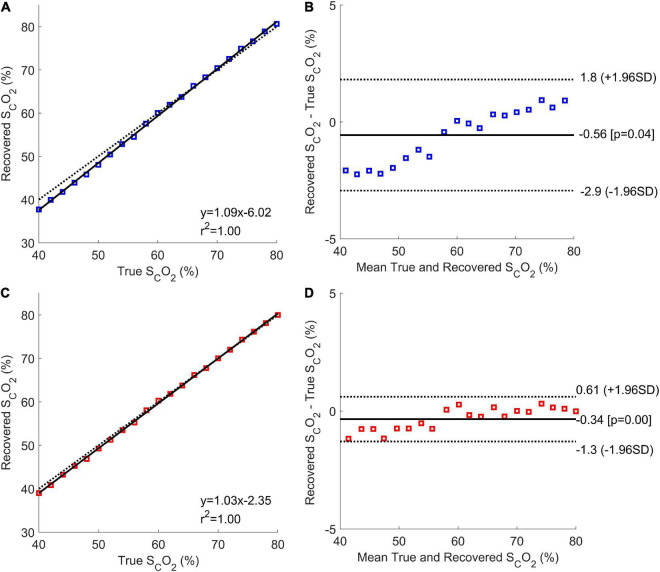
Correlation and Bland-Altman plots for the Phase 1 results with unknown (**A,B**, respectively; blue), and known (**C,D**, respectively; red) ECL thicknesses. The correlation plots show the mean estimated cerebral oxygen saturation for every true brain oxygen saturation (coloured squares), the line of best fit (solid black line), and the expected values (dotted black line).

Correlation and Bland-Altman plots analysis were conducted for both when the ECL thickness was unknown and known (Figure 6). When ECL thickness was unknown (Figure 6A), the correlation plot had a sum of squared error of 2.3% and a Pearson *R*-value squared of 1, while the correlation plot for the known ECL thickness (Figure 6C) had a sum of squared error of 1.3% and an *R*-value squared of 1. The Bland-Altman plot for the unknown ECL thickness case (Figure 6B) had a coefficient of variation of 2.0%, while the plot for the known ECL thickness (Figure 6D) had a coefficient of variation of 0.81%.

### 3.2. Phase 2: Rapid estimation of cerebral chromophore concentration

The estimated cerebral oxyhemoglobin, deoxyhemoglobin, and total hemoglobin from Phase 2 are shown in Figure 5C. The mean (± standard deviation) difference between the recovered and true brain oxygen saturation and total hemoglobin are 1.5 ± 1.5 and 3.1 ± 0.9%, respectively. Further, there is a strong agreement between the estimated and simulated values, with a Pearson’s correlation coefficient of 0.99 (*p* < 0.0001).

**FIGURE 7:**
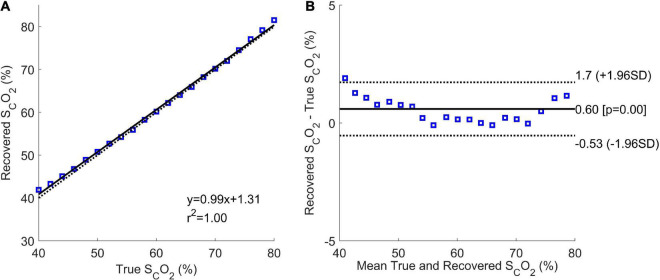
Correlation **(A)** and Bland-Altman **(B)** plots of Phase 2 results. The correlation plot shows the estimated mean cerebral oxygen saturation for every true brain oxygen saturation (blue squares), the line of best fit (solid black line), and the expected values (dotted black line).

Figure 7 shows the correlation and Bland-Altman plots for the results of Phase 2. The correlation plot had a sum of squared error of 6.2% and a Pearson *R*-value squared of 1.00. The coefficient of variation from the Bland-Altman plot was 0.96%.

## 4. Discussion and conclusion

A hyperspectral trNIRS data analysis method that can quickly and reliably estimate cerebral concentrations of oxyhemoglobin and deoxyhemoglobin was developed and validated. The findings of the current report show that the algorithm can estimate adult cerebral oxygen saturation and total hemoglobin concentration with high accuracy. Although it takes 5.2 min to analyze a full trNIRS spectrum with Phase 1, this phase is only needed once per subject to establish the baseline concentrations of oxyhemoglobin and deoxyhemoglobin. Once concentrations are established, all subsequent trNIRS spectra can be analyzed with Phase 2, which takes only 0.18 s, to estimate cerebral hemoglobin concentration and oxygen saturation. The increased speed allows for real-time neuromonitoring while maintaining high accuracy.

A major challenge when using NIRS for adult neuromonitoring is the significant signal contamination by the ECL. Previous studies have reported that the ECL is responsible for 52–88% of the detected optical signal at 2–3 cm source-detector distances, which represents a serious challenge when estimating brain chromophore concentrations (Selb et al., 2014a; Milej et al., 2020a). To investigate the potential confounding effects of the ECL on the accuracy of the method, 11 scalp blood oxygenations were simulated for every brain oxygen saturation. As shown in Figure 5, the algorithm can estimate cerebral oxyhemoglobin and deoxyhemoglobin with high accuracy despite significant changes in scalp blood oxygen saturation. The reliability of the method under a variety of ECL conditions bodes well for its use in cardiac surgery and other high-risk procedures, since the brain is considered an index organ for oxygen supply to other organs; compromised cerebral oxygen supply may be indicative of larger systemic issues (Murkin, 2011).

**FIGURE 8:**
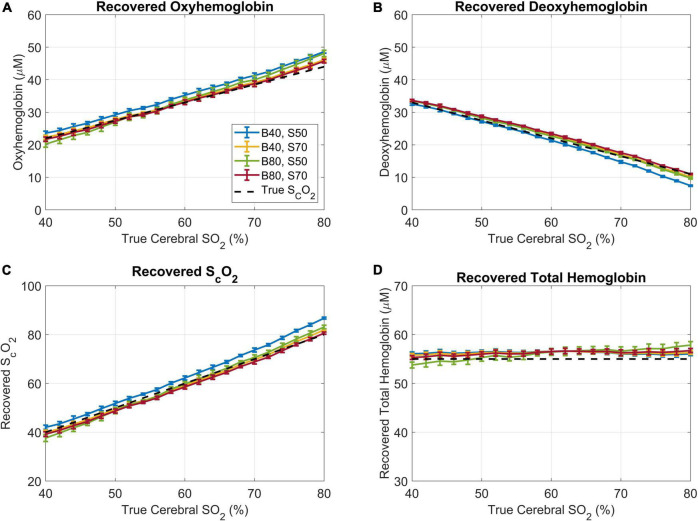
Results of Phase 2 analysis when using a wide range of brain and scalp oxygen saturations as the baseline values for estimating *f*_*ECL*_. In the legend, **(B)** denotes the simulated cerebral oxygen saturation and S represents the simulated scalp oxygenation, both in percent. **(A)** The recovered oxyhemoglobin, **(B)** recovered deoxyhemoglobin, **(C)** recovered brain oxygen saturation, and **(D)** recovered total hemoglobin plotted versus the true cerebral oxygen saturation. The dotted black lines represent the “ground truth”.

To further explore the impact of different baseline concentrations on the accuracy of Phase 2, the method was tested using the extremes of our simulated scalp and brain oxygen saturations; cerebral oxygen saturations of 40 and 80%, and scalp oxygen saturations of 50 and 70%. The analysis revealed only minor deviations with the trends remaining unchanged, and all the tested scenarios resulted in the same Pearson’s correlation coefficient of 0.99 for the recovered cerebral oxygen saturations (Figure 8). Further, a two-way ANOVA was conducted and did not reveal any statistically significant differences between the four datasets (*p* = 0.50 for the brain, and *p* = 0.49 for the scalp), showing that the fraction of optical contamination from the ECL does not significantly change with scalp or cerebral blood oxygen saturation for a given geometry within the tested range.

A major benefit of the method introduced in this report is that the exact ECL thickness does not need to be known precisely, as the fitting algorithm estimates (in Step 4) the ECL thickness within 10% of the initial guess. This could be beneficial during emergencies when there is not sufficient time for patients to receive an MRI or CT scan that would be used to estimate the ECL thickness. In such a scenario, the initial guess of the ECL thickness could be set to an average adult value as the algorithm will estimate it within 10% of the initial guess. Additionally, the relaxed requirement for the *a priori* knowledge of the ECL thickness reduces the impact of potential errors in its estimation from medical images on the accuracy of the method, as minor variations in ECL thickness are accounted for by the algorithm.

To improve robustness and reduce crosstalk, we assumed a cerebral water concentration of 80%. Since the water concentration in our simulations was 80% as well, we conducted additional analyses by assuming cerebral water concentrations of 70 and 90%. The analysis revealed that such error, which is ± 10% of the true water concentration, has a negligible impact on the accuracy of the recovered oxyhemoglobin and deoxyhemoglobin.

To reduce computational burden, Phase 1 uses a sparse spectrum (10 nm spectral resolution) instead of the dense spectrum (2 nm spectral resolution) used in Phase 2. The sparse sampling had negligible effects on accuracy; however, accuracy decreased when the spectral sampling was further reduced (i.e., more than 10 nm separation between consecutive wavelengths). Furthermore, the algorithm can be modified to recover other chromophores by increasing the number of chromophores in the fitting parameters and changing the wavelength region of interest to cover the spectral range of the target chromophores. Notably, future work will include adapting the algorithm to monitor the redox state of Cytochrome C Oxidase (CCO), a key biomarker of cerebral oxidative metabolism (Rajaram et al., 2018, 2022; Lange et al., 2019).

A potential limitation of this work is that it did not include the effects of the IRF in the Monte Carlo simulations. When analyzing trNIRS measurements, the IRF can be easily measured and its effects accounted for by convolving it with the analytical model. Note that for *hyperspectral* trNIRS measurements, the IRF of each wavelength must be measured as it has been shown that both the shape and temporal position of the IRF change with wavelength (Ioussoufovitch et al., 2021).

Furthermore, the initial rough estimates of the chromophores’ concentrations in the ECL were obtained by assuming that 2 cm SDD mainly probed the ECL. To investigate the validity of this assumption we determined the partial pathlength of all the photons detected at the 2 cm SDD in the brain and ECL, for the four extreme cases of scalp and brain oxygenation (i.e., brain oxygenation at 40 and 80% and scalp oxygenation at 50 and 70%). The analysis revealed that more than 80% of the optical pathlength of the photons detected at the 2 cm SDD are in the ECL. Therefore, the optical properties obtained by analyzing the 2 cm SDD data with a semi-infinite homogeneous model are heavily weighted toward the ECL parameters and should provide a good initial guess of the ECL optical properties.

In contrast to Steps 4 and 5, wherein all the DTOFs were fit simultaneously, Steps 1, 2, and 3 were not spectrally constrained. The authors acknowledge that spectrally constrained approaches such as the method reported by D’Andrea et al. (2006) could be used in Steps 1, 2, and 3. However, given that these estimates were only used to obtain an “educated” initial guess of the concentration of light absorbers in the ECL, which were refined in Step 4, it is reasonable to assume that applying the spectrally constrained method would not significantly alter the accuracy of the algorithm.

Another potential limitation of this work is that the simulations were conducted on one octant of the full head due to GPU memory limitations. To assess the potential impact of this approach, we randomly picked 20 simulations and analyzed the photons’ trajectories. We found that no detected photons reached the boundaries of the octant in the period of interest; thus, it is reasonable to treat the volume used in the simulations as an optically semi-infinite medium.

An additional limitation is that all validations were conducted in a single geometry. While the oxygen saturations in the scalp and brain were varied extensively, the medium was kept constant throughout. Nevertheless, we expect the algorithm to work with other ECL thicknesses since this parameter is estimated in Step 4 and thus does not need to be precisely known. Future work will include further validation in tissue-mimicking phantoms with various top layer thicknesses and in a pig model of an adult head before human applications.

In summary, this report introduces a multi-step hyperspectral trNIRS data analysis method that allows for accurate estimation of cerebral hemoglobin content and blood oxygenation in adults. Importantly, the approach does not require *a priori* knowledge of the ECL thickness, which increases robustness and usability. Further, the method can provide rapid estimates of cerebral oxygen saturation and total hemoglobin content once the baseline values are known, enabling real-time neuromonitoring. It is anticipated that this method would be valuable in a wide range of applications for continuous adult neuromonitoring, including cardiac surgery and intensive care.

## Data availability statement

The raw data supporting the conclusions of this article will be made available by the authors, without undue reservation.

## Author contributions

DC conducted the Monte Carlo simulations used for the validation and was the lead contributor to the development of the analysis method described. NL helped develop Phase 2 of the analysis method. SI provided feedback during the development of Phase 1. MD was the primary investigator for this project, providing insight and guidance throughout, as well as securing funding. All authors contributed to the article and approved the submitted version.

## References

[B1] Abdalmalak A. Milej D. Cohen D. J. F. Anazodo U. Ssali T. Diop M. (2020a). Using fMRI to investigate the potential cause of inverse oxygenation reported in fNIRS studies of motor imagery. *Neurosci. Lett.* 714:134607. 10.1016/j.neulet.2019.134607 31693928

[B2] Abdalmalak A. Milej D. Yip L. C. M. Khan A. R. Diop M. Owen A. M. (2020b). Assessing time-resolved fNIRS for brain-computer interface applications of mental communication. *Front. Neurosci.* 14:105. 10.3389/FNINS.2020.00105 32132894 PMC7040089

[B3] Auger H. Bherer L. Boucher É. Hoge R. Lesage F. Dehaes M. (2016). Quantification of extra-cerebral and cerebral hemoglobin concentrations during physical exercise using time-domain near infrared spectroscopy. *Biomed. Opt. Express* 7:3826. 10.1364/boe.7.003826 27867696 PMC5102543

[B4] Baker W. B. Balu R. He L. Kavuri V. C. Busch D. R. Amendolia O. (2019). Continuous non-invasive optical monitoring of cerebral blood flow and oxidative metabolism after acute brain injury. *J. Cerebr. Blood Flow Metab.* 39 1469–1485. 10.1177/0271678X19846657 31088234 PMC6681541

[B5] Brown D. W. Picot P. A. Naeini J. G. Springett R. Delpy D. T. Lee T. Y. (2002). Quantitative near infrared spectroscopy measurement of cerebral hemodynamics in newborn piglets. *Pediatr. Res.* 51 564–570. 10.1203/00006450-200205000-00004 11978878

[B6] Chance B. Leigh J. S. Miyake H. Smith D. S. Nioka S. Greenfeld R. (1988). Comparison of time-resolved and -unresolved measurements of deoxyhemoglobin in brain. *Proc. Natl. Acad. Sci. U. S. A.* 85 4971–4975. 10.1073/pnas.85.14.4971 3393526 PMC281669

[B7] D’Andrea C. Spinelli L. Bassi A. Giusto A. Contini D. Swartling J. (2006). Time-resolved spectrally constrained method for the quantification of chromophore concentrations and scattering parameters in diffusing media. *Opt. Express* 14:1888. 10.1364/OE.14.001888 19503518

[B8] D’Errico J. (2021). *Fminsearchbnd, fminsearchcon. MATLAB central file exchange.* Available online at: https://www.mathworks.com/matlabcentral/fileexchange/8277-fminsearchbnd-fminsearchcon (accessed on May 5, 2021).

[B9] Denault A. Y. Shaaban-Ali M. Cournoyer A. Benkreira A. Mailhot T. (2018). “Chapter 7—near-infrared spectroscopy,” in *Neuromonitoring techniques*, ed. Prabhakar H. (Amsterdam: Elsevier), 179–233. 10.1016/B978-0-12-809915-5.00007-3

[B10] Diop M. Lawrence K. S. (2013). “Deconvolution improves the accuracy and depth sensitivity of time-resolved measurements,” in *Proceedings of SPIE - the international society for optical engineering*, (Washington, DC: SPIE), 426–430. 10.1117/12.2004850

[B11] Diop M. Tichauer K. M. Elliott J. T. Migueis M. Lee T. Y. Lawrence K. S. (2010). Comparison of time-resolved and continuous-wave near-infrared techniques for measuring cerebral blood flow in piglets. *J. Biomed. Opt.* 15:057004. 10.1117/1.3488626 21054120

[B12] Diop M. Wright E. Toronov V. Lee T. Y. Lawrence K. S. (2014). Improved light collection and wavelet de-noising enable quantification of cerebral blood flow and oxygen metabolism by a low-cost, off-the-shelf spectrometer. *J. Biomed. Opt.* 19:057007. 10.1117/1.JBO.19.5.057007 24821577

[B13] Durduran T. Zhou C. Buckley E. M. Kim M. N. Yu G. Choe R. (2010). Optical measurement of cerebral hemodynamics and oxygen metabolism in neonates with congenital heart defects. *J. Biomed. Opt.* 15:037004. 10.1117/1.3425884 20615033 PMC2887915

[B14] Elliott J. T. Diop M. Tichauer K. M. Lee T. Y. Lawrence K. S. (2010). Quantitative measurement of cerebral blood flow in a juvenile porcine model by depth-resolved near-infrared spectroscopy. *J. Biomed. Opt.* 15:037014. 10.1117/1.3449579 20615043

[B15] Fang Q. Boas D. A. (2009). Monte carlo simulation of photon migration in 3D turbid media accelerated by graphics processing units. *Opt. Express* 17:20178. 10.1364/oe.17.020178 19997242 PMC2863034

[B16] Farzam P. Buckley E. M. Lin P. Y. Hagan K. Grant P. E. Inder T. E. (2017). Shedding light on the neonatal brain: Probing cerebral hemodynamics by diffuse optical spectroscopic methods. *Sci. Rep.* 7:15786. 10.1038/s41598-017-15995-1 29150648 PMC5693925

[B17] Firbank M. Hiraoka M. Essenpreis M. Delpy D. T. (1993). Measurement of the optical properties of the skull in the wavelength range 650-950 nm. *Phys. Med. Biol.* 38 503–510. 10.1088/0031-9155/38/4/002 8488176

[B18] Gagnon L. Gauthier C. Hoge R. D. Lesage F. Selb J. Boas D. A. (2008). Double-layer estimation of intra- and extracerebral hemoglobin concentration with a time-resolved system. *J. Biomed. Opt.* 13:054019. 10.1117/1.2982524 19021399 PMC2718835

[B19] Gerega A. Milej D. Weigl W. Kacprzak M. Liebert A. (2018). Multiwavelength time-resolved near-infrared spectroscopy of the adult head: Assessment of intracerebral and extracerebral absorption changes. *Biomed. Opt. Express* 9:2974. 10.1364/boe.9.002974 29984079 PMC6033559

[B20] Goldman S. Sutter F. Ferdinand F. Trace C. (2004). Optimizing intraoperative cerebral oxygen delivery using noninvasive cerebral oximetry decreases the incidence of stroke for cardiac surgical patients. *Heart Surgery Forum* 7 392–397. 10.1532/HSF98.20041062 15799908

[B21] Ioussoufovitch S. Cohen D. J. F. Milej D. Diop M. (2021). Compressed sensing time-resolved spectrometer for quantification of light absorbers in turbid media. *Biomed. Opt. Express* 12:6442. 10.1364/boe.433427 34745748 PMC8547999

[B22] Jacques S. L. (2013). Optical properties of biological tissues: A review. *Phys. Med. Biol.* 58:R37. 10.1088/0031-9155/58/11/R37 23666068

[B23] Kanti V. Bonzel A. Stroux A. Proquitté H. Bührer C. Blume-Peytavi U. (2014). Postnatal maturation of skin barrier function in premature infants. *Skin Pharmacol. Physiol.* 27 234–241. 10.1159/000354923 25059975

[B24] Kienle A. Glanzmann T. Wagnières G. van den Bergh H. (1998). Investigation of two-layered turbid media with time-resolved reflectance. *Appl. Opt.* 37:6852. 10.1364/ao.37.006852 18301502

[B25] Kikinis R. Pieper S. D. Vosburgh K. G. (2014). “3D Slicer: A Platform for Subject-Specific Image Analysis, Visualization, and Clinical Support,” in *Intraoperative imaging and image-guided therapy*, ed. Jolesz F. (New York, NY: Springer), 277–289. 10.1007/978-1-4614-7657-3_19

[B26] Lange F. Dunne L. Hale L. Tachtsidis I. (2019). MAESTROS: A multiwavelength time-domain nirs system to monitor changes in oxygenation and oxidation state of cytochrome-C-oxidase. *IEEE J. Select. Top. Quantum Electron.* 25:7100312. 10.1109/JSTQE.2018.2833205 30450021 PMC6054019

[B27] Lewis C. Parulkar S. D. Bebawy J. Sherwani S. Hogue C. W. (2018). Cerebral neuromonitoring during cardiac surgery: A critical appraisal with an emphasis on near-infrared spectroscopy. *J. Cardiothorac. Vasc. Anesth.* 32 2313–2322. 10.1053/j.jvca.2018.03.032 30100271

[B28] Li N. C. Diop M. (2022). “Analysis of Near-Infrared Spectroscopy Measures of Cerebral Oxygen Metabolism in Infants,” in *Proceedings biophotonics congress: Biomedical optics 2022 (translational, microscopy, OCT, OTS, brain)*, (Washington, DC: Optica Publishing Group), 10.1364/TRANSLATIONAL.2022.JM3A.60

[B29] Li Z. Park B. K. Liu W. Zhang J. Reed M. P. Rupp J. D. (2015). A statistical skull geometry model for children 0-3 years old. *PLoS One* 10:e0127322. 10.1371/JOURNAL.PONE.0127322 25992998 PMC4436309

[B30] Liebert A. Wabnitz H. Grosenick D. Möller M. Macdonald R. Rinneberg H. (2003). Evaluation of optical properties of highly scattering media by moments of distributions of times of flight of photons. *Appl. Opt.* 42: 5785. 10.1364/ao.42.005785 14528944

[B31] Luttkus A. Fengler T. W. Friedmann W. Dudenhausen J. W. (1995). Continuous monitoring of fetal oxygen saturation by pulse oximetry. *Obst. Gynecol.* 85 183–186. 10.1016/0029-7844(94)00353-F 7529914

[B32] Milej D. Shahid M. Abdalmalak A. Rajaram A. Diop M. Lawrence K. S. (2020b). Characterizing dynamic cerebral vascular reactivity using a hybrid system combining time-resolved near-infrared and diffuse correlation spectroscopy. *Biomed. Opt. Express* 11 4571–4585. 10.1364/BOE.392113 32923065 PMC7449704

[B33] Milej D. Abdalmalak A. Rajaram A. Lawrence K. (2020a). Direct assessment of extracerebral signal contamination on optical measurements of cerebral blood flow, oxygenation, and metabolism. *Neurophotonics* 7:045002. 10.1117/1.nph.7.4.045002 33062801 PMC7540337

[B34] Murkin J. M. (2011). Cerebral oximetry: Monitoring the brain as the index organ. *Anesthesiology* 114 12–13. 10.1097/ALN.0b013e3181fef5d2 21178667

[B35] Oros-Peusquens A. M. Loução R. Abbas Z. Gras V. Zimmermann M. Shah N. J. (2019). A single-scan, rapid whole-brain protocol for quantitative water content mapping with neurobiological implications. *Front. Neurol.* 10:1333. 10.3389/FNEUR.2019.01333 31920951 PMC6934004

[B36] Patterson M. S. Chance B. Wilson B. C. (1989). Time resolved reflectance and transmittance for the noninvasive measurement of tissue optical properties. *Appl. Opt.* 28:2331. 10.1364/ao.28.002331 20555520

[B37] Rajaram A. Bale G. Kewin M. Morrison L. B. Tachtsidis I. Lawrence K. (2018). Simultaneous monitoring of cerebral perfusion and cytochrome c oxidase by combining broadband near-infrared spectroscopy and diffuse correlation spectroscopy. *Biomed. Opt. Express* 9:2588. 10.1364/boe.9.002588 30258675 PMC6154190

[B38] Rajaram A. Milej D. Suwalski M. Kebaya L. Kewin M. Yip L. (2022). Assessing cerebral blood flow, oxygenation and cytochrome c oxidase stability in preterm infants during the first 3 days after birth. *Sci. Rep.* 12:181. 10.1038/s41598-021-03830-7 34996949 PMC8741949

[B39] Scholkmann F. Kleiser S. Metz A. Zimmermann R. Mata P. J. Wolf U. (2014). A review on continuous wave functional near-infrared spectroscopy and imaging instrumentation and methodology. *Neuroimage* 85 6–27. 10.1016/j.neuroimage.2013.05.004 23684868

[B40] Selb J. Ogden T. M. Dubb J. Fang Q. Boas D. A. (2014b). Comparison of a layered slab and an atlas head model for Monte Carlo fitting of time-domain near-infrared spectroscopy data of the adult head. *J. Biomed. Opt.* 19:016010. 10.1117/1.JBO.19.1.016010 24407503 PMC3886581

[B41] Selb J. Boas D. A. Chan S. T. Evans K. C. Buckley E. M. Carp S. A. (2014a). Sensitivity of near-infrared spectroscopy and diffuse correlation spectroscopy to brain hemodynamics: Simulations and experimental findings during hypercapnia. *Neurophotonics* 1:015005. 10.1117/1.NPh.1.1.015005 25453036 PMC4247161

[B42] Selb J. Stott J. J. Franceschini M. A. Sorensen A. G. Boas D. A. (2005). Improved sensitivity to cerebral hemodynamics during brain activation with a time-gated optical system: Analytical model and experimental validation. *J. Biomed. Opt.* 10:011013. 10.1117/1.1852553 15847579

[B43] Sharma S. D. Park E. Purcell P. L. Gordon K. A. Papsin B. C. Cushing S. L. (2020). Age-related variability in pediatric scalp thickness: Implications for auditory prostheses. *Int. J. Pediatr. Otorhinolaryngol.* 130:109853. 10.1016/J.IJPORL.2019.109853 31887567

[B44] Steinbrink J. Fischer T. Kuppe H. Hetzer R. Uludag K. Obrig H. (2006). Relevance of depth resolution for cerebral blood flow monitoring by near-infrared spectroscopic bolus tracking during cardiopulmonary bypass. *J. Thoracic Cardiovasc. Surg.* 132 1172–1178. 10.1016/j.jtcvs.2006.05.065 17059940

[B45] Wojtkiewicz S. Sawosz P. Milej D. Treszczanowicz J. Liebert A. (2014). “Development of a Multidistance Continuous Wave Near-Infrared Spectroscopy Device with Frequency Coding,” in *Biomedical optics 2014 Paper BM3A.24*, (Washington, DC: The Optical Society), 10.1364/biomed.2014.bm3a.24

[B46] Yan S. Fang Q. (2020). Hybrid mesh and voxel based Monte Carlo algorithm for accurate and efficient photon transport modeling in complex bio-tissues. *Biomed. Opt. Express* 11:6262. 10.1364/boe.409468 33282488 PMC7687934

